# Live Imaging of Antifungal Activity by Human Primary Neutrophils and Monocytes in Response to *A. fumigatus*

**DOI:** 10.3791/55444

**Published:** 2017-04-19

**Authors:** Shan F. Brunel, Jude M. Bain, Jill King, Lena J. Heung, Shinji Kasahara, Tobias M. Hohl, Adilia Warris

**Affiliations:** ^1^Aberdeen Fungal Group, MRC Centre for Medical Mycology, Institute of Medical Sciences, University of Aberdeen; ^2^Department of Medicine, Memorial Sloan-Kettering Cancer Center, New York, US

**Keywords:** Immunology, Issue 122, *Aspergillus fumigatus*, live cell imaging, antifungal activity, FLARE, human leucocytes, host-pathogen interaction.

## Abstract

*Aspergillus fumigatus* is an opportunistic fungal pathogen causing invasive infections in immunocompromised hosts with a high case-fatality rate. Research investigating immunological responses against *A. fumigatus* has been limited by the lack of consistent and reliable assays for measuring the antifungal activity of specific immune cells *in vitro*. A new method is described to assess the antifungal activity of primary monocytes and neutrophils from human donors against *A. fumigatus* using FLuorescent *Aspergillus* REporter (FLARE) conidia. These conidia contain a genetically encoded dsRed reporter, which is constitutively expressed by live FLARE conidia, and are externally labeled with Alexa Fluor 633, which is resistant to degradation within the phagolysosome, thus allowing a distinction between live and dead *A. fumigatus* conidia. Video microscopy and flow cytometry are subsequently used to visualize the interaction between conidia and innate immune cells, assessing fungicidal activity whilst also providing a wealth of information on phagocyte migration, phagocytosis and the inhibition of fungal growth. This novel technique has already provided exciting new insights into the host-pathogen interaction of primary immune cells against *A. fumigatus*. It is important to note the laboratory setup required to perform this assay, including the necessary microscopy and flow cytometry facilities, and the capacity to work with human donor blood and genetically manipulated fungi. However, this assay is capable of generating large amounts of data and can reveal detailed insights into the antifungal response. This protocol has successfully been used to study the host-pathogen interaction of primary immune cells against *A. fumigatus*.

It is important to note the laboratory setup required to perform this assay, including the necessary microscopy and flow cytometry facilities, and the capacity to work with human donor blood and genetically manipulated fungi. However, this assay is capable of generating large amounts of data and can reveal detailed insights into the antifungal response. This protocol has successfully been used to study the host-pathogen interaction of primary immune cells against *A. fumigatus*.

**Figure:**
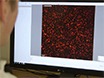


## Introduction

*Aspergillus fumigatus *is an opportunistic fungal pathogen and the most common fungal cause of invasive lung infections in the immunocompromised host^1^,^2^. Understanding how host immune cells recognize and eliminate *Aspergillus *is an important area of fungal research, however, the currently available fungicidal assays have significant limitations. Commonly used methods of measuring fungicidal activity include colorimetric assays and determination of colony forming units^3^,^4^. However, such methods provide information on fungal viability at a single time point rather than viewing the dynamic interaction between host and pathogen. Accordingly, these methods cannot take into account whether a fungus has been killed or simply (temporarily) restricted in growth or metabolic activity. We have developed a method capable of observing fungicidal activity directly whilst simultaneously capturing detailed information regarding phagocytosis, phagocyte migration and inhibition of fungal growth.

Previously, a protocol was published using real-time imaging as a means to measure phagocytosis of *Candida albicans* by a mouse macrophage cell line^5^. This concept has now been developed further to address the knowledge gap in our understanding of *Aspergillus* interactions with immune cells by utilizing FLuorescent *Aspergillus* REporter (FLARE) conidia^6^,^7^,^8^. These conidia express a red fluorophore (dsRed) and are labeled with a second fluorophore (Alexa Fluor 633). Once a FLARE conidium is killed, it stops producing dsRed and the remaining dsRed fades, while the AF633 remains fluorescent and bound to the conidia. This creates a clear distinction between live and dead fungal cells during live cell imaging and flow cytometry, and enables tracking of the fate of individual conidia following their interaction with immune cells.

The described assays provide an effective method of visualizing the interaction between host immune cells and *Aspergillus* and will be of considerable value in unravelling the cellular signaling pathways responsible for antifungal effector mechanisms in innate immune cells. Other applications include visualizing how changes in *Aspergillus* growth, such as the switch from resting to swollen conidia, or from swollen conidia to hyphae, affect phagocyte recognition and function.

The following protocol describes in detail how to obtain human monocytes and neutrophils; how to prepare the FLARE conidia; and how to capture their responses with video microscopy and flow cytometry. Although the use of human immune cells isolated from donor blood is described here, this technique has proven equally effective using a variety of murine cell populations.

## Protocol

The protocol for obtaining neutrophils and peripheral blood mononuclear cells (PBMC) is based on previously published methods^9^. The use of blood samples from healthy volunteers has been approved by the human research ethics committee of the University of Aberdeen. Before starting make sure all the ethical approvals are in place for drawing blood from healthy volunteers and/or patients for the purpose of the described experiment. Keep cells on ice where possible to increase their survival.

### 1. *A. fumigatus* Culture and Conditions

1. Prepare glucose minimal agar medium as described^10^.
  1. Adjust media to pH 6.5 using 1 M NaOH and autoclave at 120 °C for 20 min.
  2. Allow to cool to 65 °C.
  3. Working in a sterile flow hood, pour 30 mL of cooled media into a T75 flask and allow to cool with the neck of the flask resting on a 25 mL pipette to create an agar slope.
2. Streak out the dsRed Af293-*A. fumigatus* strain on the agar and incubate for 7 days at 37 °C + 5% CO_2_. Two flasks will yield approximately 10^9 ^conidia in 5 mL phosphate-buffered saline (PBS) pre-biotinylation.

### 2. *A. fumigatus* Labeling and Preparation

Note: When performing this assay for the first time, prepare the parental Af293-*A. fumigatus* strain. Take samples of both strains in microcentrifuge tubes and label only one of each strain as described below. This will allow one to have control conidia with no color and conidia with a single color, either dsRed or AF633, to test the setup and equipment.

1. Harvest *A. fumigatus* conidia by immersing the culture with 30 mL PBS + 0.05% Tween-80. Filter the resulting suspension through a 40 µm cell strainer into a 50 mL tube to remove hyphal fragments.
2. Centrifuge at 805 x g for 10 min, remove the supernatant and wash once in 20 mL of sterile PBS. Pool conidia from two plates of the same strain during the wash step.
3. Following the washing step, resuspend the pellet in 5 mL of PBS and transfer 1 mL aliquots of conidial suspension into microcentrifuge tubes. 1 mL of suspension of each strain is usually sufficient for live cell imaging. Wrap the remaining aliquots in foil and store at 4 °C.
4. Centrifuge aliquots of conidial suspension at 9,300 x g for 10 min at room temperature and carefully remove supernatant. Resuspend the conidial pellet in 1 mL 0.05 M NaHCO_3_ pH 8.3.
5. Prepare 10 mg biotin/200 µL dimethylsulfoxide (DMSO) stock solution by reconstituting 25 mg biotin in 500 µL DMSO. Add 10 µL biotin/DMSO stock solution to the microcentrifuge tube, cover in aluminum foil and incubate for 2 h on a rocker at 4 °C.
  1. Aliquot the remainder of the biotin/DMSO stock solution and freeze at -20 °C for use in subsequent experiments.
6. Centrifuge for 10 min at 9,300 x g and carefully remove the supernatant. Resuspend the conidial pellet in 1 mL of 100 mM Tris-HCl pH 8.0 for 1 h to deactivate free-floating biotin.
7. Centrifuge for 10 min at 9,300 x g and carefully remove the supernatant. Wash the pellet twice with 1 mL of sterile PBS and resuspend the pellet in 1 mL of PBS.
8. Dissolve 1 mg of Streptavidin-AF633 in 0.5 mL of PBS to make a 2 mg/mL stock solution. Add 10 µL of 2 mg/mL streptavidin-AF633 per 1 mL of conidial suspension and incubate for 40 min at room temperature on a rocker covered in aluminum foil. The remaining Streptavidin-AF633 can be frozen in aliquots for use in subsequent experiments.
9. Centrifuge for 10 min at 9,300 x g and carefully remove supernatant. Resuspend the conidial pellet in 1 mL of PBS and count the conidia using a hemocytometer at a 1:1,000 dilution (1:100 and then 1:10). Adjust the conidial concentration to 3.6 x 10^6^/mL with CO_2_ independent media, wrap in foil and store at 4 °C. NOTE: The conidia are now labeled with AF633 and will be referred to as FLARE conidia.
10. Optional*:* If stimulation with swollen conidia is required, after counting conidia (step 2.9), dilute conidia to 7.2 x 10^6^/mL in yeast nitrogen base (YNB) medium and add 500 µL conidial suspension to 4.5 mL YNB medium in an autoclaved 50 mL Erlenmeyer flask. Cover and place the flask in a shaking incubator (200 rpm at 37 °C) for 6 h.
  1. Transfer the medium to a 15 mL tube. Centrifuge at 805 x g for 10 min at room temperature and wash once in PBS. Centrifuge again and resuspend pellet in 1 mL CO_2_ independent medium, giving 3.6 x 10^6^ conidia/mL. *Note:* Conidia frequently clump after they become swollen making accurate counting difficult, it is advised to calculate with the counted numbers of resting conidia used.

### 3. Isolation of Human Neutrophils and Monocytes

1. Draw 20 mL of venous blood from healthy volunteers into two 10 mL blood tubes containing ethylenediaminetetraacetic acid (EDTA). For each donor separately, pour 20 mL blood in a 50 mL tube and dilute with 15 mL PBS.
2. Underlay the blood with 15 mL of a lymphocyte isolation solution (density 1.077 g/mL) with a syringe and iron needle. Place the needle at the bottom of the tube and gently force the lymphocyte isolation solution out. Centrifuge at 630 x g for 20 min with no brake and low acceleration.
3. Prepare PBS, chilled on ice. NOTE: Steps 3.4 and 3.5 must be followed simultaneously to generate monocytes (3.4) and neutrophils (3.5).
4. Using a pastette, harvest the PBMC layer. This is the layer in the center between the yellow serum (upper) and the transparent lymphocyte isolation solution (lower) layer, and transfer into a fresh 50 mL tube.
  1. Fill the tube with the PBMC up to 50 mL with cold PBS and centrifuge at 582 x g for 10 min. Remove the supernatant and wash twice with cold PBS and resuspend in 1 mL of PBS per 20 mL of donor blood used initially.
  2. Make 1:10 dilutions for counting by adding 20 µL of the cell suspension to 160 µL of PBS and 20 µL of trypan blue. Count cells with a hemocytometer.
  3. Prepare a buffer solution by adding 26 mL of heat inactivated fetal calf serum (FCS) and 2.1 mL of 0.5 M EDTA to 500 mL of HBSS. Centrifuge the cells for 10 min at 515 x g and resuspend in 40 µL of buffer solution per 1 x 10^7^ cells.
  4. Transfer the cell suspension to a 15 mL tube and add 10 µL of CD14 microbeads per 1 x 10^7^ cells, mix well and incubate for 15 min at 4 °C, making sure to mix the tubes every 5 min.
  5. Wash cells by adding 1 mL of buffer solution per 1 x 10^7^ cells and centrifuge at 515 x g for 10 min.
  6. Aspirate supernatant completely and resuspend up to 1 x 10^8^ cells in 500 µL of buffer solution.
  7. Place 1 column per sample on a magnetic separator according to the manufacturer's instructions. First wash the column once with 500 µL of buffer solution.
  8. Pipette the 500 µL of cell suspension through the column, wait for the column to dry and then wash by repeating this three times with buffer solution.
  9. Place a new 15 mL tube underneath the column and take the column off the magnetic separator. Then flush the cells out of the column into the tube with 1 mL of buffer solution.
  10. Add 4 mL of buffer solution and centrifuge at 515 x g for 10 min, and then resuspend in 1 mL of Roswell Park Memorial Institute (RPMI) medium.
  11. Make 1:10 dilutions for counting by adding 20 µL of the cell suspension to 160 µL of PBS and 20 µL of trypan blue. Count the cells with a hemocytometer.
  12. Adjust the cell concentration to 6 x 10^5^/mL with RPMI and seed 200 µL on a 35 mm glass-based imaging dish. Incubate overnight at 37 °C with 5% CO_2_.
  13. The next day, prior to imaging, remove the RPMI and add 200 µL of CO_2_ independent medium. Note: These monocytes can also be differentiated into various macrophage subsets prior to imaging. If this is a technique to be explored, an excellent starting point is the recent paper by Ohradanova-Repic *et al.**1*1.
5. After harvesting the PBMC layer, remove all of the serum and most of the lymphocyte isolation solution, but be careful not to remove the small white band on top of the red pellet as this will contain most of the neutrophils.
  1. Prepare a 10x hypotonic lysis buffer stock by adding 83 g of NH_4_Cl and 10 g of KHCO_3_ in 1 L of sterile water. Store at 4 °C.
  2. Dilute this stock 10x with sterile water and add it to the tubes. Then, carefully invert 3 times and incubate in ice for 15 min.
  3. Centrifuge at 394 x g for 10 min and resuspend in hypotonic lysis buffer for 10 min in ice.
  4. Centrifuge at 394 x g and wash twice with cold PBS. Resuspend in 1 mL of CO_2_ independent medium per donor.
  5. Make 1:10 dilutions for counting by adding 20 µL of the cell suspension to 160 µL of PBS and 20 µL of trypan blue. Count the cells with a hemocytometer.
  6. For flow cytometry, adjust the concentration to 6 x 10^5^/mL and add 400 µL cell suspension to a 24-well plate. For microscopy, add 200 µL of the same cell suspension to a 35 mm glass-based imaging dish. Note*:* Neutrophil imaging or flow cytometry should be initiated immediately due to their propensity to undergo apoptosis if left unstimulated. If this is not possible neutrophils should be kept on ice and used as soon as possible (within the same day).

### 4. Flow Cytometry

1. Add 200 µL of 1.2 x 10^6^/mL FLARE conidia to the cells in the 24-well plate, and then add 100 µL of 20 µg/mL voriconazole. Add 300 µL of RPMI and incubate for 16 h at 37 °C with 5% CO_2_. NOTE: Voriconazole is added to prevent conidial germination.
2. Add 25 µL of a 1 mM Calcofluor-white stock solution to each well for a final concentration of 25 µM and incubate for 10 min at 37 °C with 5% CO_2_.
3. Centrifuge at 582 x g for 10 min. Remove the supernatant and wash twice with PBS.
4. To harvest adherent cells (monocytes), add 1 mL of PBS with 3 mM EDTA to each well and incubate for 10 min at 37 °C with 5% CO_2_. Gently wash cells off the plate by pipetting and collect cells in a tube.
5. Wash once in FACS Buffer (2% fetal calf serum to PBS), and then resuspend in FACS Buffer for FACS analysis.
  1. For FACS analysis, first adjust the compensation parameters of the flow cytometer and set positive gates using single-colored compensation tubes, including conidia with no color: dsRed+ conidia, AF633+ conidia, and Calcofluor White+ conidia (generated as in 4.2).
  2. Set free conidia gate based on forward and side scatter using conidia with no color. Set cell gate on forward and side scatter by excluding the free conidia gate.
  3. Analyze sample tube by recording 10,000 events. NOTE: Cells associated with live conidia will be dsRed+ and AF633+. Cells associated with dead conidia will be AF633+ only. Bystander cells that have not engaged conidia will be dsRed- and AF633-. The conidia-engaged cell populations are further analyzed for Calcofluor White staining on the Pacific Blue channel. Conidia that remain outside of cells will be Calcofluor White+, and conidia that have been internalized will be Calcofluor White-.

### 5. Live Cell Video Microscopy

Note: The choice of microscope will depend upon what is available locally, but the microscope setup will need to include an inverted stage, an environmental chamber heated to 37 °C and appropriate excitation/emission filters for dsRed (532/561 nm) and AF633 (633 nm).**FLARE conidia do not work with the 488 nm laser in lieu of the 532/561 nm laser for dsRed. When performing this experiment for this first time, use the control conidia with no color and single color to test for background fluorescence and bleed-through between the dsRed and AF633 channels.

1. Turn on the microscope heater prior to the experiment and allow sufficient time for the environmental control chamber to warm to 37 °C. The time taken for chamber temperature to stabilize will vary for different microscope setups.
2. Turn on the microscope and computer, and load the imaging software. Mount the imaging dish on the microscope stage and adjust the focus to find the human neutrophils or monocytes.
3. For dsRed and AF633, set the laser power to 10% and exposure time to 1 s in the acquisition settings.
4. Remove the imaging slide and add 100 µL of 3 x 10^6^/mL FLARE conidial suspension to the appropriate wells (total volume 300 µL/well). Record the time that FLARE conidia are added to the dish.
5. Return the slide to the stage and adjust camera sensitivity for dsRed and AF633 to clearly see the conidia but not overexpose the image. Set up a stage points list if multi-point acquisition is required.
6. Commence imaging when all points are in focus, the channels are optimized and the cycle time and duration is set. Cycle time and duration of imaging depend on the experimental question. The goal is to keep the cycle time as short as possible (≤2 min) with 1 min allowing for in depth analysis of uptake dynamics.

## Representative Results

Representative results are shown following the described protocol. **Figure 1** illustrates a representative 6 h live-cell video with human neutrophils and FLARE conidia. dsRed (red) is expressed by the conidia themselves while they have been labeled with AF633 (Magenta).

**Figure 2** is an image from a single time point from a similar movie as displayed in **Figure 1**, illustrating the difference between live and dead FLARE conidia. Dead conidia are distinguished from live conidia by losing their dsRed (red) signal while maintaining a bright AF633 (magenta) fluorescence. *A. fumigatus* conidia often survive within phagocytes for over 6 h. Therefore, to effectively quantify killing, flow cytometry plots are shown for a 16 h incubation period in **Figure 3**. This data was obtained with an alveolar macrophage cell line as an example but can be performed with any cell type. In **Figure 4** the migration is shown in the first hour of stimulation for human neutrophils as well as their velocity and directional movement. **Figure 5** shows the percentage of neutrophils and monocytes that have ingested 1, 2, 3 or more conidia while **Figure 6** displays the number of conidia that germinate into hyphae.


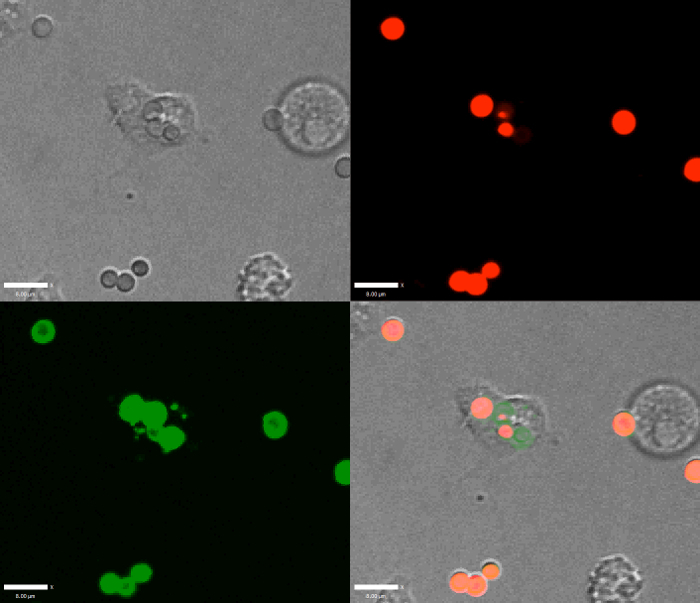
**Figure 1: Live-cell video microscopy movie of human neutrophils and FLARE conidia. **Neutrophils were isolated from human blood and seeded together with FLARE conidia in CO_2_ independent medium at a ratio of 1:3, respectively. Imaging was initiated directly at 37 °C with a spinning disk confocal microscope. Images were captured at 1 min and 55 s intervals over a period of 6 h. Scale bar = 10 µm. A zoomed in video is shown with the channels separated to clarify the role of the FLARE conidia with DIC in the top left, dsRed (red) in the top right, AF633 (green) in the bottom left and the merged video in the bottom right. Please click here to download this video.


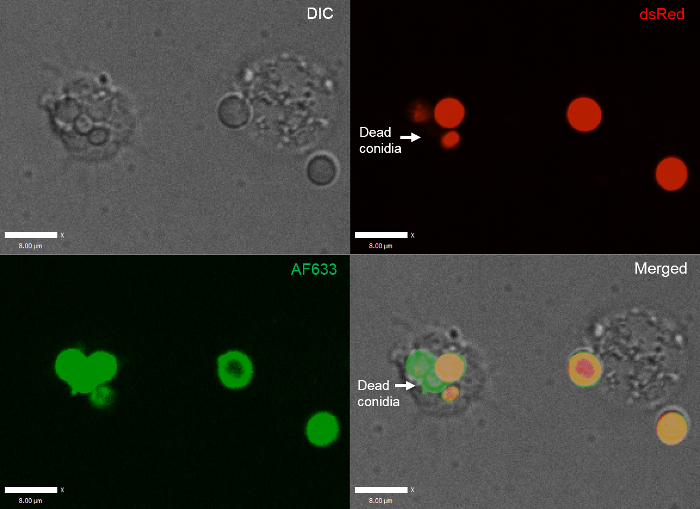
**Figure 2: Single time point image demonstrating the difference between live and dead FLARE conidia. **Human neutrophils were stimulated with FLARE conidia and imaged with a spinning disk confocal microscope. An image was taken from the generated movies. Dead conidia are distinguished from hyphae by having lost their dsRed (red: top right) signal while maintaining their AF633 fluorescence (green: bottom left). Please click here to view a larger version of this figure.


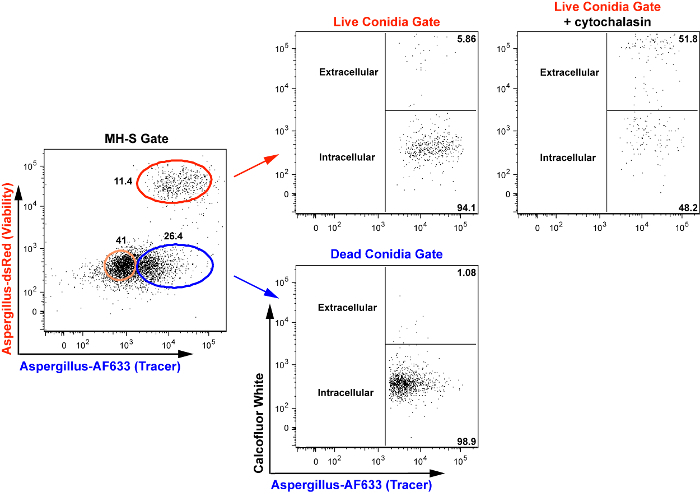
**Figure 3: Quantification and validation of phagocytosis and killing of *A. fumigatus* conidia. **Mouse alveolar macrophage cells (MH-S) were incubated with FLARE conidia at a 1:1 ratio for 16 h in the presence of voriconazole. MH-S cells associated with live conidia are shown in the red gate (dsRed+AF633+). MH-S cells associated with dead conidia are shown in the blue gate (dsRed-AF633+). Bystander MH-S cells are shown in the gold gate. Calcofluor White, a cell-impermeable fluorescent dye that binds to the fungal cell wall, is used to distinguish extracellular conidia (Calcofluor White+) from intracellular conidia (Calcofluor White-). As an extra validation to the method, it is shown that treatment with 2 µM cytochalasin D inhibits conidial uptake by MH-S cells. Please click here to view a larger version of this figure.


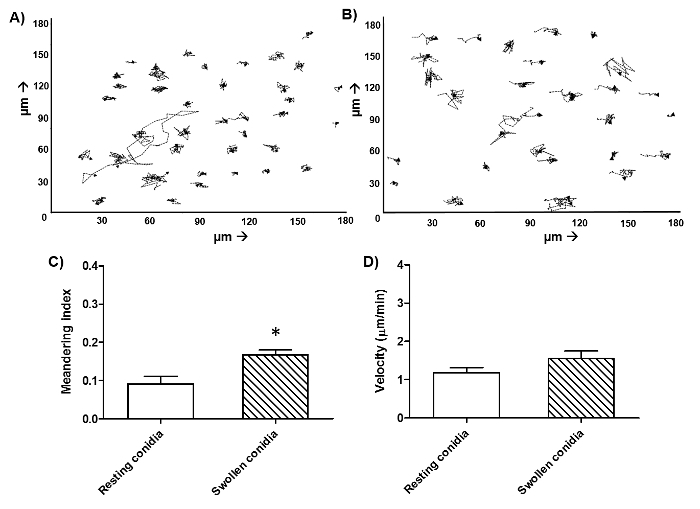
**Figure 4: Migration of human neutrophils and monocytes against *A. fumigatus* conidia. **Neutrophils were isolated from human blood and seeded together with FLARE conidia in CO_2_ independent medium at a ratio of 1:3, respectively. Imaging was initiated directly at 37 °C with a spinning disk confocal microscope. Images were captured at 1 min and 55 s intervals over a period of 6 hours. Migration of all individual human neutrophils per field was manually tracked using tracking software against resting (**A**) and swollen (**B**) conidia for 1 h. Data represents 1 frame of cells for each condition of the same donor. The meandering factor (**C**), a measure of directional movement, and velocity (**D**) were then quantified using the same software. Mean ± SEM for 3 donors are shown with 30 cells per donor over 2 independent experiments. *: p < 0.05 (Welch's corrected t test). Please click here to view a larger version of this figure.


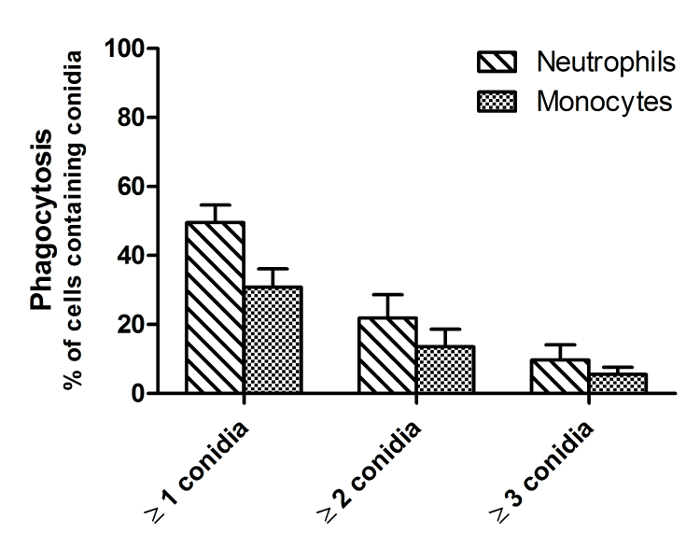
**Figure 5: Phagocytosis of *A. fumigatus* conidia by human neutrophils and monocytes. **Neutrophils and monocytes were isolated from human blood and seeded together with FLARE conidia in CO_2_ independent medium at a ratio of 1:3, respectively. Imaging was initiated directly at 37 °C with a spinning disk confocal microscope. Images were captured at 1 min and 55 s intervals over a period of 6 h. Phagocytosis was measured as the amount of cells containing FLARE conidia after 4 h of stimulation. Data is presented as mean ± SEM from 3 donors and 3 frames per donor over 2 independent experiments. Please click here to view a larger version of this figure.


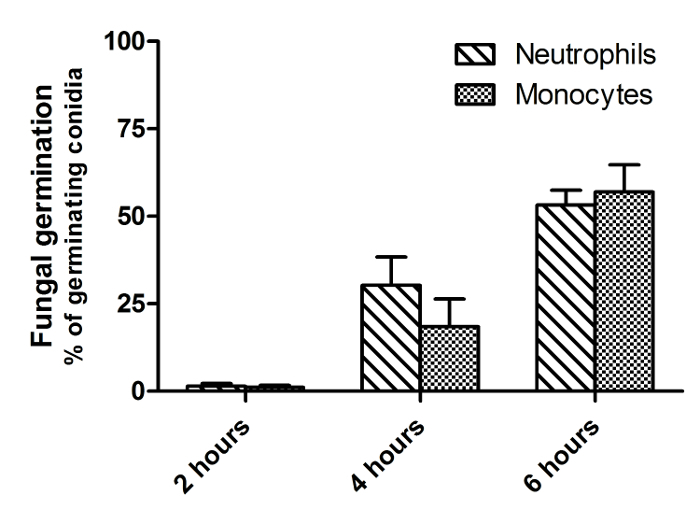
**Figure 6: Inhibition of germination of *****A. fumigatus***** conidia by neutrophils and monocytes. **Neutrophils and monocytes were isolated from human blood and seeded together with FLARE conidia in CO_2_ independent medium at a ratio of 1:3, respectively. Imaging was initiated directly at 37 °C with a spinning disk confocal microscope. Images were captured at 1 min and 55 s intervals over a period of 6 hours. Inhibition of fungal germination was examined by measuring the percentage of conidia that had germinated after 2, 4 and 6 h of co-culture. Data is presented as mean ± SEM from 3 donors and 3 frames per donor over 2 independent experiments. Please click here to view a larger version of this figure.

## Discussion

This method describes an innovative* in vitro *method to study the antifungal activity of human primary phagocytes against *A. fumigatus *using FLuorescent *Aspergillus* REporter (FLARE) conidia, live cell imaging and flow cytometry. Previous studies have demonstrated the added value of using FLARE conidia both *in vivo* in murine experimental fungal infection and *in vitro* assessment of antifungal immunity^6^,^7^. Combining FLARE conidia with this next generation live cell imaging technique allows for a setup to measure viability of individual conidia during dynamic interactions *in vitro*.

The described method has the potential to investigate the specific roles and importance of certain cellular processes of immune cells on their antifungal responses. In addition, antifungal responses of phagocytes from specific patients suffering from defined single component defects in their immune system can be assessed in greater detail. Very early and initial antifungal responses can be studied in great detail over 6 h of continuous imaging. This allows for even subtle differences to be detected, such as the velocity of phagocyte migration towards the fungus, internalization rates, dynamics of fungicidal events^12^, which would be undistinguishable using conventional phagocytosis methods.

Any cell type could be used with this method; the cell types used here were selected because these phagocytes constitute the first and second lines of defense in the human airways against *A. fumigatus*^13^. Interestingly, very clear differences can be observed between how monocytes and neutrophils engage with resting and swollen conidia and hyphae. Monocytes hardly migrate to conidia, while neutrophils display much more migratory activity. Additional fluorescent markers such as live-dead markers on immune cells can be included in the assay to provide insights into phagocyte survival following interactions with *Aspergillus*. Interesting potential future applications for this technology include the co-culture of different host cell types, the consequences for antifungal responses (*e.g.*, macrophages on an epithelial monolayer, monocyte-derived dendritic cells together with neutrophils), and the real-time measurement of reactive oxygen species production or neutrophil extracellular trap formation following stimulation with *Aspergillus*.

A few points need to be noted to use this protocol, foremost the laboratory setup required: a microscope with an inverted stage, an environmental chamber stable at 37 °C, and excitation/emission filters for dsRed (532/561 nm) and AF633 (633 nm). The ability of the microscope to keep the cells in focus and retain the oil immersion (particularly relevant during multi-point acquisition) should be monitored periodically due to the long duration of imaging. For quantification of fungicidal activity flow cytometry facilities are required. Additionally, appropriate institutional and ethical approvals need to be in place to work with healthy donor and patient derived blood samples and genetically manipulated fungi. It is strongly recommended to use fresh blood samples (use on the same day) to increase cell viability and preserve functionality. Ideally, isolation of cells is performed immediately after drawing the blood samples and neutrophils are imaged immediately after isolation.

In conclusion, a next generation live cell imaging technique to assess in great detail phagocyte antifungal activity against *A. fumigatus *is described. This technology can provide a unique insight into individual cell-fungal interactions in early innate immune defenses against *Aspergillus*.

## Disclosures

The authors have nothing to disclose.
